# The current use and attitudes towards tumor genome sequencing in breast cancer

**DOI:** 10.1038/srep22517

**Published:** 2016-03-02

**Authors:** I. Gingras, A. Sonnenblick, E. de Azambuja, M. Paesmans, S. Delaloge, Philippe Aftimos, M. J. Piccart, C. Sotiriou, M. Ignatiadis, H. A. Azim

**Affiliations:** 1 Department of Hematology and Oncology, Hôpital du Sacré-Coeur de Montréal, Montreal, Quebec, Canada; 2 Department of Medicine, BrEAST Data Centre, Institut Jules Bordet/Université libre de Bruxelles, Brussels, Belgium; 3 Breast Cancer Translational Research Laboratory J. C. Heuson, Institut Jules Bordet/Université libre de Bruxelles, Brussels, Belgium; 4 Medical Oncology Clinic, Institut Jules Bordet/Université libre de Bruxelles, Brussels, Belgium; 5 Data Centre, Institut Jules Bordet/Université libre de Bruxelles, Brussels, Belgium; 6 Department of Medical Oncology, Institut Gustave-Roussy, Villejuif, France

## Abstract

There is increasing availability of technologies that can interrogate the genomic landscape of an individual tumor; however, their impact on daily practice remains uncertain. We conducted a 28-item survey to investigate the current attitudes towards the integration of tumor genome sequencing in breast cancer management. A link to the survey was communicated via newsletters of several oncological societies, and dedicated mailing by academic research groups. Multivariable logistic regression modeling was carried out to determine the relationship between predictors and outcomes. 215 physicians participated to the survey. The majority were medical oncologists (88%), practicing in Europe (70%) and working in academic institutions (66%). Tumor genome sequencing was requested by 82 participants (38%), of whom 21% reported low confidence in their genomic knowledge, and 56% considered tumor genome sequencing to be poorly accessible. In multivariable analysis, having time allocated to research (OR 3.37, 95% CI 1.84–6.15, *p* < 0.0001), working in Asia (OR 5.76, 95% CI 1.57 – 21.15, *p* = 0.01) and having institutional guidelines for molecular sequencing (OR 2.09, 95% 0.99–4.42, *p* = 0.05) were associated with a higher probability of use. In conclusion, our survey indicates that tumor genome sequencing is sometimes used, albeit not widely, in guiding management of breast cancer patients.

Precision medicine is a rising paradigm in cancer care. Emerging technologies are now allowing for comprehensive molecular characterization of cancer at the individual gene level, with increasing efficacy and at ever-lower price^1^. Genome sequencing can identify potential driver mutations that could be candidate for targeted therapies^2^. However, the implementation of genotype-driven oncology has uncovered the many challenges associated with a broader use of genome sequencing platforms, such as the uncertainties regarding the true biological significance of each alteration, potential heterogeneity between the primary tumor and metastatic deposits, the unintended discovery of germ-line mutations of unknown significance, and the rather low access to drugs targeting the identified alterations^3^,^4^,^5^,^6^.

In breast cancer, the genomic-driven approach, that is, the selection of drugs matching tumor molecular alterations to treat patients with cancer^7^, has yet to demonstrate superiority to standard of care beyond targeting the estrogen receptor (ER) and HER2 pathways. However, emerging pre-clinical and clinical evidences suggest potential benefits of this approach^8^,^9^,^10^,^11^,^12^. Anecdotal cases of patients experiencing exceptional response to a targeted therapy have been reported in different cancer types^13^,^14^,^15^. Recent results from non-randomized clinical trials evaluating the feasibility of a genotype-driven treatment approach in advanced breast cancer suggest that potentially “actionable” genomic alterations can be identified in a significant proportion of breast cancer patients^8^,^9^,^12^,^16^. However, there is no robust evidence that these genomic alterations are valid predictive biomarkers to determine benefit from a given targeted agent^17^, and evidence suggest that the predictive value of a genomic alteration may not be the same across different tumor types^18^. Recently, the results of the first randomized trial comparing the benefit of treatment selected according to the tumor molecular profile to standard treatment (SHIVA trial) were published^19^. The study failed to demonstrate increased progression-free survival with the molecular-driven approach.

Evidences addressing current practices and beliefs of oncologists regarding multiplex tumor genome sequencing are limited^20^,^21^,^22^. Previous studies suggest that being a medical oncologist, having used cancer genome sequencing in the past and having a high degree of confidence in making genetic recommendations are factors associated with the increased rate of using these tests^20^,^21^. However, none of these studies addressed practices specifically in breast cancer patients. Thus, we conducted a survey to examine the current attitude among oncologists towards the integration of tumor genome sequencing platforms in the clinical management of breast cancer patients.

## Results

### Participants’ characteristics

Two hundred and fifteen physicians from 35 countries completed the online survey between the 9^th^ of March and the 30^th^ of June 2015, with 199/215 (93%) fully completed questionnaires. Eighty-eight percent of the participants were medical oncologists. The majority of participants were working in Europe (70.2%), the most frequent countries reported being Italy (35/215, 16%), Belgium (25/215, 12%), and France (22/215, 10%). Two-third of the participants were working in an academic institution, and 40% had more than 25% of their time allocated to research. The participants were experienced in following breast cancer patients, with more than 90% of participants seeing at least six new breast cancer patients per month (Table 1). Only 40/215 (19%) of participants confirmed that there were guidelines in their institute for the use of multiplex tumor genome sequencing for breast cancer patients.

### Current use of tumor genome sequencing

Eighty-two of the 215 participants (38%) had used tumor genome sequencing for their breast cancer patients at least once in the past. In multivariable logistic regression analysis, there was a statistically significant association between using tumor genome sequencing and having more than 25% of time allocated to research (OR 3.37, 95% CI 1.84–6.15, *p* < 0.0001), working in Asia (OR 5.76, 95% CI 1.57–21.15, *p* = 0.01) and having institutional guidelines for molecular sequencing (OR 2.09, 95% 0.99–4.42, *p* = 0.05) (Table 2).

**Figure 1:**
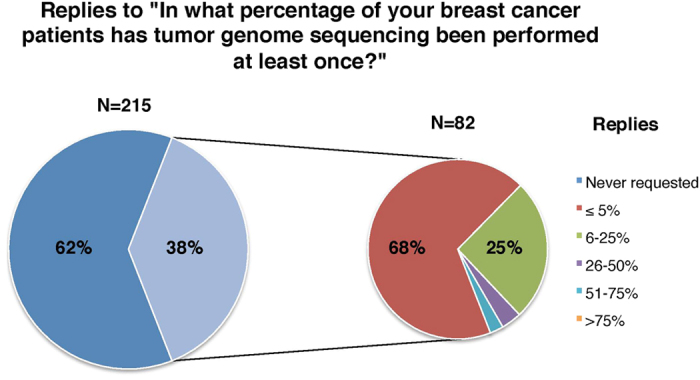
Participants’ use of tumor genome sequencing.

**Figure 2:**
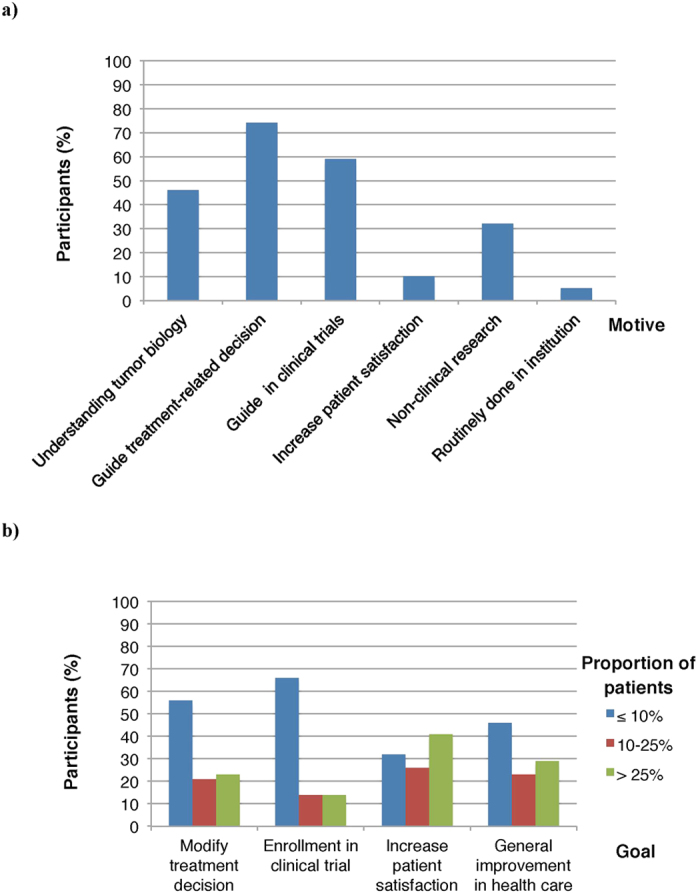
Participants’ (**a**) motive(s) to request tumor genome sequencing for breast cancer patients, and (**b**) perception of the proportion of patients for which these goals are achieved.

**Figure 3:**
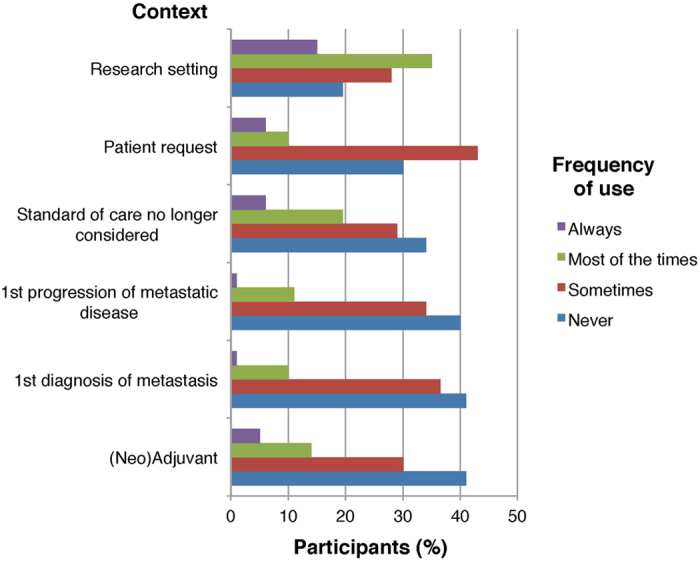
Participants’ frequency of uses of tumor genome sequencing in various contexts.

Of the 82 participants that had used tumor genome sequencing in the past, 49/82 (60%) had used it equal or less than 5 times during the past 6 months, and 56/82 (68%) indicated that such testing was performed in equal or less than 5% of their breast cancer patients (Fig. 1). Although most of the participants stated that they requested tumor genome sequencing to guide treatment-related decisions (61/82, 74%) or for the potential enrollment of patients in clinical trials (49/82, 60%), the majority reported that this is achieved in less than 10% of patients (Fig. 2a,b). When questioned about the probability to use tumor genome sequencing in various clinical contexts, sequencing was more likely to be requested “most of the times” or “always” when standard treatments were no longer considered (21/82, 25%) or in the research setting (41/82, 50%) (Fig. 3).

### Degree of confidence

Most of the participants affirmed to be “somewhat” (43/82, 52%), or “highly” (22/82, 27%) confident in interpreting tumor genome sequencing results. However, 36/82 (44%) declared that support to interpret the results was insufficient. Half of the participants (42/82, 51%) affirmed that a tumor board dedicated to molecular screening was organized in their institution. In univariate and multivariate analysis, there was a statistically significant association between having a high degree of confidence and having a tumor board dedicated to molecular screening in institution (OR 3.62, 95% CI 1.12–11.66, *p* = 0.03) as well as with seeing more new breast cancer patients per months (OR 4.54, 95% CI 1.30–15.84, *p* = 0.02) (Table 3).

### Accessibility and funding

Principal sources of funding for tumor genome sequencing were research funds (39/82, 48%), self-funded patients (26/82, 32%), and patient private health insurance (24/82, 29%). Most of the responders affirmed using commercial sequencing platforms accredited either in Europe (30/82, 37%) or in the US (27/82, 33%). More than half (46/82, 56%) considered tumor genome sequencing to be “not at all” or “poorly” accessible.

**Figure 4:**
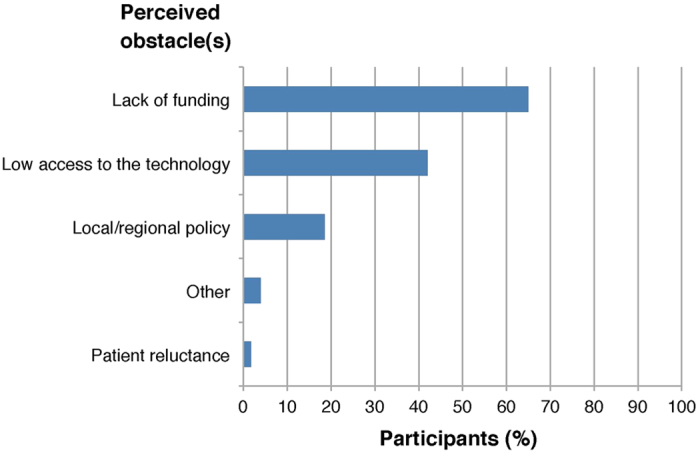
Participants’ perceived obstacles to request tumor molecular sequencing for breast cancer patients.

The 215 participants were questioned on the perceived obstacle(s) to request tumor genome profiling in daily clinical practice, and the majority (140/215, 65%) highlighted the lack of funding (Fig. 4). When asked if, provided that tumor genome sequencing would become more accessible, they would request for sequencing more often, most of the participants replied “Yes” (58.5%) or “maybe” (34%), while only 7.5% replied that they would not. The majority of participants (191/215, 89%) thought that tumor genome sequencing will play a central role in the management of breast cancer patients in the short future. The most frequent concerns to use tumor genome sequencing in daily clinical practice were the lack of evidence of benefit (129/215, 60%), and the lack of access to corresponding clinical trials (113/215, 53%).

## Discussion

In this survey, we endeavored to interrogate the current use of multiplex tumor genome sequencing for breast cancer patients. Our study suggests that tumor genome sequencing is sometimes used, albeit not commonly, in the clinical management of breast cancer patients. This study is the first to suggest that having more time allocated to research is associated with an increased probability of using tumor genome sequencing. Greater accessibility to sequencing platforms, better access to corresponding clinical trials and increased awareness of the literature in the field could explain why oncologists involved in research would use these platforms more widely. Our study also suggests that tumor genome sequencing use varies according to geographic area, with more frequent use in Asian countries. This finding has to be interpreted with caution, given the low number of participants that were working outside of Europe. Differences in accessibility, especially in funding resources, may possibly explain the results. Past studies have demonstrated that being a medical oncologist (compared to surgeon oncologist) is associated with more use of tumor genome sequencing^20^,^21^; in our study, the vast majority of the participants were medical oncologists (88%), therefore we were not able to confirm this association. Because questions about confidence and accessibility were only addressed to participants that had used tumor genome sequencing in the past, we could not verify the association between these variables and the probability of tumor genome sequencing use.

The results of our survey suggest that in clinical practice, the proportion of breast cancer patients in which the results of tumor genome sequencing lead to modifying treatment decision or enrolling patients in specific clinical trials remains low, which is in line with previous studies in the field^9^,^16^,^23^,^24^. In the feasibility part of the SHIVA trial, 28 patients had advanced breast cancer^16^; of these, only five (5/28, 18%) had an actionable genomic aberration (*PI3KCA* mutation or *PTEN* loss) identified by high-throughput genome sequencing. In the SAFIR-01 trial, out of the 407 metastatic breast cancer patients enrolled, molecular screening was possible in 297 patients (70%), with an identified genetic alteration in 172 (42%); however, only 55 (13%) ultimately received tailored treatment^9^. Finally, in the MOSCATO-01 trial, of the first 129 breast cancer patients enrolled, an actionable target was identified in 52 patients (40%), and only 25 patients (23%) could be treated with a corresponding targeted therapy^23^. Although these studies demonstrated the feasibility of the genotype-driven approach, they underscored that only a fraction of patients can ultimately receive tailored treatment; the same conclusion was reached following a large scale genomic testing initiative across tumor types including 2,000 patients, of which 39% had an “actionable mutation”, but only 11% went on genotype-matched trials^24^. The SHIVA trial failed to demonstrate benefit of the molecular-driven approach compared to standard treatment; The MOSCATO-01 trials is still ongoing and could provide evidence on the benefit of a genotype-driven approach specifically in breast cancer. We also expect that large and longitudinal international initiatives such as AURORA will serve the goal of discovery as well as clinical relevance of genome sequencing in breast cancer^25^.

Our survey suggest little consensus on the context in which tumor genome sequencing should be requested. Only 19% of participants had specific guidelines for the use of tumor genome sequencing in their institution. Although recommendations for the use of genomic signature for prognostic evaluation in early breast cancer has been issued by the American Society of Clinical Oncology (ASCO), the Evaluation of Genomic Applications in Practice and Prevention (EGAPP) working group, the IMPAKT 2012 working group and ESMO^26^,^27^,^28^,^29^, no recommendations on the use of multiplex tumor genome sequencing to guide treatment-related decision in advanced breast cancer have been issued so far. In the future, validation of new predictive biomarkers may prove the relevance of breast cancer genome sequencing in routine practice. For example, while ER is currently the only biomarker guiding endocrine treatment in breast cancer, the discovery of *ESR1* mutations and their prediction of treatment resistance will probably play an increasing role in treatment decisions^30^,^31^. Thus it is plausible that other alterations with predictive value for response to targeted therapies might also emerge^32^. Genomic information as part of the medical file of the patient may become necessary for enrollment in basket and umbrella trials^33^. However, clinical trials are needed to evaluate the true capacity of different genomic alterations to predict benefit from targeted therapy. Early implementation of tumor molecular profiling tools in clinical practice before reaching sufficient level of evidence may impair future research to demonstrate clinical benefit. Therefore, we would advocate restricting the use of multiplex tumor genome profiling to the research setting, until robust demonstration of clinical utility is achieved.

In our study, 21% of the participants declared to have poor confidence in interpreting tumor genome sequencing results, which compares to the degree of confidence reported in the study by Gray and colleagues that investigated the use multiplex somatic genomic testing in a single cancer center, before the initiation of an enterprise-wide multiplex testing initiative^20^. Our results suggest an association between the presence of an institutional tumor board dedicated to molecular screening and a high degree of confidence to interpret genome-sequencing results. Many studies have explored the confidence of physicians towards the use of genomic tests to predict the susceptibility to develop different types of cancer, such as BRCA-associated breast cancer^34^,^35^. Although a large body of evidence exist to support the use of some of these tests such as BRCA mutation screening, these studies consistently suggested that improvement in the health care providers’ knowledge and confidence about genomic testing is highly needed^22^,^34^,^35^,^36^,^37^,^38^. These studies also suggested that educational program can improve the confidence level of the physicians^39^. The results of our study and previous studies in the field are supporting the development of dedicated tumor boards and educational programs to help clinicians with the interpretation of genomic data if molecular sequencing platforms are to be more broadly used in the future. Recently, the ASCO launched the Molecular Oncology Tumor Boards series, a user-driven resource designed to help the health care providers with the interpretation of molecular profiling tests.

We found that accessibility to tumor genome sequencing remains generally low, mainly because of the lack of funding. This is not surprising since, in the absence of concrete evidence of clinical utility, many of the public health care systems do not reimburse such testing. In the study by Gray and colleagues, 25% of the oncologists anticipated using tumor genome sequencing in ≥90% of their patients, which is considerably higher than what is reported in our study^20^. However, unlike in our study, the participants of the Gray study were all working in the same academic institution and expected very high accessibility to tumor genome profiling because of the anticipated implementation of a broad genomic profiling initiative in their center.

Our survey has few drawbacks that should be pointed out. The survey was communicated via the ESMO and ESO newsletters and by dedicated mailing by academic groups. The ESMO newsletter is transmitted to over 13,000 members from 130 different countries. We are expecting that only a small fraction of members acknowledged the link to the survey; however, true response rate cannot be accurately estimated, and may be low. Therefore, self-selection bias and sampling bias cannot be excluded. However, the method used to distribute the survey allowed for oncologist from different backgrounds and parts of the world to participate, and thus to interrogate for the first time the differences in the use of tumor genome sequencing according to these variables.

Because the survey was distributed primarily by academic organizations, it is likely that oncologists working in an academic institution and having time allocated to research were overrepresented in our study compared to the general oncology community. Since these factors are associated with increased use of tumor genome sequencing, it is plausible that the frequency of use in the general oncology community is lower than what is reported in this study. Moreover, physicians with more interest in genomic science may have been more inclined to answer the survey. The majority of participants were from European countries (70%), therefore the results of this study may not represent practices outside of Europe. Finally, the questions used in our survey to measure different parameters such as confidence or accessibility were not previously validated in other trials. However, it is worth noting that this is one of the very first efforts to understand the potential applicability and use of these tools in breast cancer practice and we believe could serve as an importance resource to understand the challenges in this field.

In conclusion, our study highlights that lack of evidence is still a major concern for wider use of a genotype-driven approach in breast cancer. As evidence grows to support precision medicine in breast cancer, more guidance will be needed to support physicians in optimizing the use of molecular profiling tools in daily practice.

## Methods

### Survey instrument

We conducted an online 28-item survey consisting of multiple choice and Likert-scale questions (see Supplementary Table S1). A short introduction specified that the survey was not addressing the use of gene expression profiling assays that evaluate the risk of recurrence in early breast cancer, such as OncotypeDx®, Mammaprint® and Prosigna®.

The first six questions captured demographic data and characteristics of the participants. The remaining 22 questions assessed different aspects of the use of tumor genome sequencing including frequency of use, context and reasons to use tumor genome sequencing, perceived utility of such testing, confidence in interpreting the results, accessibility and funding, and future perspectives. Skip logic was applied for questions 8–23 so that responders that had never used tumor genome sequencing in the past would not answer these questions.

### Survey dissemination

A web link to the online survey was communicated via various platforms such as the European Society for Medical Oncology (ESMO) and European School of Oncology (ESO) newsletters, and via a dedicated mailing by the Breast International Group (BIG) and other academic research groups including the Belgian Society of Medical Oncology (BSMO), the French Breast Cancer Intergroup UNICANCER (UCBG) and the Associazione Italiana di Oncologia Medica (AIOM) Giovani. Completion of the survey was anonymous and no honorarium was provided to any of the study participants to complete the survey.

### Statistical analysis

Descriptive analysis was undertaken using Excel software from Microsoft Office. Multivariable logistic regression modeling was carried out to determine the relationship between predictors and either the use of tumor genome sequencing (Yes or No) or the expressed degree of confidence to interpret the results (high or not) using SAS software. Univariate analyses were conducted first and used to make a pre-selection of covariates to be tested in a multivariable setting. Only covariates that showed a p value < 0.30 in the univariate model were considered for the multivariable model. For use of genome sequencing, a stepwise backward selection of covariates was applied to select the final model while a forward stepwise method was used for the other binary outcomes. Odds ratios were estimated and are reported with 95% confidence intervals and their statistical significance was tested using exact probability distribution. All hypothesis testing were based on two-tailed tests with *P* value of less than 0.05.

## Additional Information

**How to cite this article**: Gingras, I. *et al*. The current use and attitudes towards tumor genome sequencing in breast cancer. *Sci. Rep*. **6**, 22517; doi: 10.1038/srep22517 (2016).

## Supplementary Material

Supplementary Information

## Figures and Tables

**Table 1:** Participants’ characteristics (N = 215).

Characteristics		# of participants (%)
Age	Median	43
	Range	27–77
# Years of clinical practice	0–10	82 (38.1)
	11–20	66 (30.7)
	21–30	44 (20.5)
	>30	23 (10.7)
Field of practice	Medical oncology	189 (88)
	Other*	26 (12)
Continent of clinical practice	Africa	4 (1.9)
	Asia	12 (5.6)
	Europe	151 (70.2)
	North America	7 (3.3)
	South America	22 (10.2)
	Oceania	16 (7.4)
	Missing	3 (1.4)
Working environment	Academic	141 (65.6)
	Non-Academic	74 (34.4)
Proportion of time allocated to research	0–25%	128 (59.5)
	26–50%	68 (31.6)
	>50%	19 (8.9)
Number new breast cancer patients/months	<5	21 (9.7)
	6–10	58 (27.0)
	11–25	61 (28.4)
	>25	75 (34.9)

^*^Includes hemato-oncology, gyneco-oncology, pathology, radiation oncology, surgical oncology.

**Table 2:** Logistic regression model to predict use of tumor genome sequencing (n = 215).

Variables		Univariate analysis OR (95% CI)		*P* Value	Multivariate analysis* Adjusted OR (95% CI)		*P* Value
Academic institution (*vs* Non-Academic)		2.33	(1.21–4.60)	0.006			
Time allocated to research (>25% *vs* ≤25%)		2.85	(1.56–5.28)	0.0002	3.37	1.84–6.15	<0.0001
Field (Other *vs* Medical Oncologist)		1.02	(0.39–2.53)	0.97			
Number of new breast cancer patient/months (>10 *vs* ≤10)		1.43	(0.77–2.67)	0.23			
Years of clinical practice (>10 *vs* ≤10)		1.11	(0.61–2.05)	0.71			
Continent of clinical practice (*vs* Europe)	Asia	3.56	(0.90–16.93)	0.07	5.76	1.57–21.15	0.01
	Other continents	1.27	(0.63–2.55)	0.50	1.39	(0.68–2.82)	0.37
Guidelines in institute (Yes *vs* No/I do not know)		2.06	(0.97–4.39)	0.06	2.09	0.99–4.42	0.05

^*^Results showed only for variables associated with use of tumor genome sequencing with *p* value equal or less to 0.05.

**Table 3:** Logistic regression model to predict high degree of confidence* (n = 82).

Variables	Univariate analysis OR (95% CI)		*P* Value	Multivariate analysis** Adjusted OR (95% CI)		*P* Value
Academic institution (vs Non-Academic)	3.90	(0.80–38.05)	0.08			
Time allocated to research (>25% vs ≤25%)	2.64	(0.84–9.40)	0.07			
Field (Other vs Medical Oncologist)	1.98	(0.37–9.49)	0.32			
Number of new breast cancer patient/months (>10 vs ≤10)	6.05	(1.60–24.84)	0.003	4.54	1.30–15.84	0.02
Years of clinical practice (>10 vs ≤10)	0.47	(0.15–1.42)	0.21			
Dedicated tumor board (Yes vs No)	4.67	(1.41–18.40)	0.01	3.62	1.12–11.66	0.03
Frequency of use in the past 6 months (>5 vs ≤5)	2.85	(0.94–8.99)	0.07			
Guidelines in institute (Yes *vs* No/I do not know)	2.04	(0.61–6.70)	0.29			

^*^The modeled probability is the probability of being highly confident.

^**^Results showed only for variables associated with high degree of confidence with *p* value equal or less to 0.05.
