# Supplementary material for: The current use and attitudes towards tumor genome sequencing in breast cancer
Source: Sci Rep. 2016 Mar 2;6:22517. doi: 10.1038/srep22517 (PMC4773754; doi:10.1038/srep22517)
Supplement: Supplementary Information [file srep22517-s1.pdf]

The current use and attitudes towards tumor genome sequencing in breast cancer

I. Gingras[1], MD; A. Sonnenblick[1,2], MD, PhD; E. de Azambuja[1,3], MD, PhD; M. Paesmans[4], M. Sc.; S. Delaloge[5] , MD; Philippe Aftimos[3], MD; M. J. Piccart[1,3], MD, PhD; C. Sotiriou[2,3], MD, PhD; M. Ignatiadis[2,3], MD, PhD; H. A. Azim Jr[1,2], MD, PhD.

1. Department of Medicine, BrEAST Data Centre, Institut Jules Bordet/Université libre de Bruxelles, Brussels, Belgium;

2. Breast Cancer Translational Research Laboratory J. C. Heuson, Institut Jules Bordet/Université libre de Bruxelles, Brussels, Belgium

3. Medical Oncology Clinic, Institut Jules Bordet/Université libre de Bruxelles, Brussels, Belgium

4. Data Centre, Institut Jules Bordet/Université libre de Bruxelles, Brussels, Belgium

5. Department of Medical Oncology, Institut Gustave-Roussy, Villejuif, France

Corresponding author:

Isabelle Gingras, MD

Institut Jules Bordet

BrEAST Data Centre

Boulevard de Waterloo, 121

1000 Brussels,Belgium

Tel: ++32.2/541.30.99

E-mail: isabelle.gingras@bordet.be

Supplementary Table S1: 28 questions included in the online survey

Number	Question
**1**	Years of clinical practice as an oncologist?
**2**	Current country of clinical practice?
**3**	Is your current practice mostly based in?<br>[Academic versus non-academic institution]
**4**	Proportion of time allocated to research?
**5**	Field of specialization?
**6**	Average number of new breast cancer patients per month in your clinic?
**7**	Have you ever used cancer genome sequencing techniques (excluding genomic assays that are only assessing the risk of recurrence in ER-positive breast cancer patients like OncotypeDx, Mammaprint, Prosigna, etc…) for clinical decision-making in breast cancer patients in your practice?
**8**	In the past 6 months, how many times have you asked for such test?
**9**	In what percentage of your breast cancer patients has tumor genome sequencing been performed at least once?
**10**	In which context(s) do you typically request for tumor genome sequencing in breast cancer patients?<br>[(Neo)adjuvant, 1st diagnosis of metastasis, 1st progression of metastatic disease. Standard of care no longer considered, patient request, research setting]
**11**	What is (are) your main reason(s) to request for tumor genome sequencing in breast cancer patients?
**12**	How often do you modify your treatment decision according to results?
**13**	How often do the results lead to the enrollment of the patient in a specific clinical trial?
**14**	How often do you believe the test increase patient's satisfaction with health care?
**15**	How often do you believe these tests result in general improvement of patient care?
**16**	How confident are you in interpreting tumor sequencing results?
**17**	Do you believe that support to interpret tumor genome sequencing is sufficient and accessible?
**18**	Are tumor boards dedicated to molecular screening organized in your institute?
**19**	Where do you seek support and information when you experience difficulty with interpreting tumor sequencing results?
**20**	What is (are) the principal source(s) of funding for tumor genome sequencing in your region?
**21**	What is (are) the main platform(s) to perform tumor genome sequencing in your institute?
**22**	If you are using commercial platform(s), please specify which one
**23**	In your routine clinical practice, you consider that tumor genome sequencing is [Accessibility]
**24**	What is (are) the principal obstacle (s) to the use of tumor genome testing for breast cancer patients in your routine clinical practice?
**25**	If tumor genome sequencing were more accessible, would you request it more often

	in your routine clinical practice?
**26**	In your institute, is there local/regional guidelines concerning personalized medicine and the request for tumor genome sequencing in the management of breast cancer patients?
**27**	Do you believe that personalized medicine and tumor genome sequencing will play a central role in the management of breast cancer patients in the next 5-10 years?
**28**	What are your principal concerns about the use of tumor genome sequencing in routine clinical practice?
